# *Astragalus membranaceus* Has Potential Anti-Aging and Anticancer Effects on Skin and Bone

**DOI:** 10.3390/biom16060864

**Published:** 2026-06-12

**Authors:** Zainab R. Abdelrahman, Amani A. Harb, Shtaywy S. Abdalla

**Affiliations:** 1 Department of Biological Sciences, School of Science, The University of Jordan, Amman 11942, Jordan; whitelilium10@gmail.com; 2 Department of Allied Sciences, Faculty of Arts and Sciences, Al-Ahliyya Amman University, Amman 19111, Jordan

**Keywords:** *Astragalus membranaceus*, skin aging, osteoporosis, melanoma, osteosarcoma, TA65, astragalosides, cycloastragenol

## Abstract

*Astragalus membranaceus*, a medicinal plant used in traditional Chinese medicine for centuries, has attracted growing scientific attention for its potential anti-aging and anticancer properties, particularly for skin and bone health. Its key bioactive compounds like astragalosides, cycloastragenol, and its commercial derivative TA-65, have been associated with telomerase activation and telomere maintenance, suggesting a possible role in modulating cellular senescence and tissue repair processes. In addition to the claimed telomere maintenance, *A. membranaceus* exhibits antioxidant, anti-inflammatory, and DNA-protective activities, properties that contribute to its anti-aging effects. Emerging evidence also suggests that telomerase modulation by *A. membranaceus* influences cancer cell dynamics, either suppressing tumor progression through immune regulation and apoptosis induction or, in some contexts, potentially promoting tumor growth. This duality highlights the importance of dose, formulation, and targeted application. Clinically, TA-65 has been reported to improve vascular health, bone mineral density, and skin elasticity in aging individuals. Preclinical studies further support its protective effects against osteoporotic bone loss and photoaging-induced dermal degeneration. This review summarizes the phytochemical composition of *A. membranaceus* and critically evaluates the mechanistic and therapeutic evidence underlying its anti-aging and anticancer actions on skin and bone tissues. It also discusses the pharmacokinetic properties of *A. membranaceus*, including its absorption, bioavailability, and safety profile. The integration of *A. membranaceus* into evidence-based senile therapeutic strategies holds promise, but further mechanistic and clinical studies are required to optimize its safety and efficacy.

## 1. Introduction

The genus *Astragalus* (family Leguminosae, Fabaceae) comprises approximately 2900 species globally, with *Astragalus membranaceus* (Fisch.) (syn. *A. mongholicus*) Bunge being one of the most widely studied medicinal species [1]. Commonly known as Huangqi, its dried root (*Astragali Radix*) has been used in traditional Chinese medicine for over 2000 years in formulations designed to enhance vitality, strengthen immunity, and delay aging [1,2]. It is still being used in various herbal preparations. The marketed preparation, TA-65, which contains the bioactive compound cycloastragenol (CAG), has been commercially used as a dietary supplement [3]. It has been reported to exhibit telomerase-activating properties and to improve blood pressure disturbance, bone density, and metabolic markers in healthy individuals [3].

The aim of this review is to provide a comprehensive overview of the phytochemistry and the pharmacological activities of *A. membranaceus*, with emphasis on its anti-aging and anticancer effects on skin and bone, and to discuss its translational potential in nutraceutical, cosmetic, and clinical applications. This work specifically addresses a gap in the literature by focusing on the effects of *A. membranaceus* on skin (melanoma) and bone (osteosarcoma) cancers, which have received limited experimental and analytical attention. Complementing previous reviews that briefly touched on these cancers within broader discussions of antitumor activity, this study provides a dedicated and structured synthesis of the available evidence. In addition, it offers an integrated overview of the underlying molecular mechanisms, thereby updating current knowledge and highlighting the therapeutic potential of *A. membranaceus* in both anticancer and anti-aging applications for skin and bone malignancies. Furthermore, the pharmacokinetics of *A. membranaceus*, including bioavailability and safety, is discussed to improve understanding and optimize its efficacy.

## 2. Phytochemistry

Phytochemical analyses reveal that *A. membranaceus* is rich in saponins, flavonoids, polysaccharides, amino acids, alkaloids, and other secondary metabolites [3]. More than 200 compounds have been identified, including over 100 flavonoid derivatives [1]. For example, Jung et al. reported the isolation from the hexane and ethyl acetate extracts, four triterpenoids (lupenone, friedelin, lupeol, and soyasapogenol E), and seven sterols: β-sitosterol, stigmastane-3,6-dione, 7α-hydroxysitosterol, 5α,6β-dihydroxysitosterol, 7-oxo-β-sitosterol, β-sitosterol glucoside 6′-O-palmitate, and β-sitosterol glucoside [4]. Also, from the 70% ethanolic extract of *A. membranaceus* roots, Lee et al. reported that eleven flavonoid derivatives and a lignan glycoside were isolated and identified by spectroscopic methods, including liquiritigenin, daidzein, formononetin, sophorophenolone, calycosin, methylnissolin, isomucronulatol, isomucronulatol 7-O-glucoside, methylnissolin 3-O-glucoside, calycosin 7-O-glucoside, (+)-syringaresinol O-β-D-glucoside, and isomucronulatol 7,2′-di-O-glucoside [5]. Other flavones (kaempferol, isorhamnetin, rhamnocitrin, kumatakenin, rhamnocitrin-3-glucoside, and quercetin-3-glucoside) and isoflavones (formononetin, ononin, calycosin, calycosin-7-O-β-D-glucoside-6″-O-malonate, 3′-methoxy-5′-hydroxy-isoflavone-7-O-β-D-glucoside, and (3R)-2′,3′-dihydroxy-4′,7-dimethoxyisoflavone) have been isolated from *A. membranaceus* [6]. Earlier, Kitagawa (1983) reported the isolation of cycloartane triterpenoids such as astragaloside I–VIII and isoastragalosides I and II, whereas several other astragalosides (VII and VIII) have been identified as saponins with an oleanane skeleton [7]. Astragalus saponins like isoastragalosides III and IV, astramembrannin II, cyclogaleginoside B, cycloaraloside A, brachyoside B, cyclocanthoside E, cyclounifolioside B, and astramembranosides A and B have also been reported [6]. Other saponins have also been identified in the plant, including astragaloside I, Isoastragaloside II, astragaloside II, agroastragaloside I, cyclogaleginoside B, cycloaraloside A, brachyoside B, agroastragaloside II, astragaloside III, astragaloside IV, astramembranoside A, astramembranoside B, cylocanthoside E, cyclounifolioside B, and azukisaponin V methyl ester [8].

As for polysaccharides, more than 30 types have been isolated from *Astragalus*, which include dextrans and heteropolysaccharides [9]. Additionally, rhamnose, xylose, glucose, galactose, mannose, and alcohol-soluble polysaccharide, which is composed of mannose, glucose, galactose, and arabinose with pyranose rings and α-glycosidic bonds, have been identified [10]. Moreover, the following elements have been identified: molybdenum, copper, manganese, scandium, rubidium, selenium, chromium, cobalt, cesium, iron, and zinc, and more than 20 trace elements, with iron, manganese, zinc, and aluminum contents higher than those of the others [11]. Furthermore, about 20 amino acids, including arginine, aspartic acid, asparagine, proline, and alanine, have been identified [12]. Furthermore, other compounds such as folic acid, palmitic acid, bitter elements, coumarin, chloric acid, coumaric acid, choline, linolenic acid, legume sterols, ferulic acid, isoferulic acid, hydroxyphenyl acrylic acid, deerskinol, betaine, caffeic acid, linoleic acid, and β-sitosterol have also been identified from *Astragalus* species. It should be mentioned, however, that three toxic phytoconstituents, namely indolizidine alkaloids (trihydroxyoctahydroindolizidine), aliphatic nitro compounds, and iron–selenium derivatives, have been also discovered [6,13].

**Figure 1:**
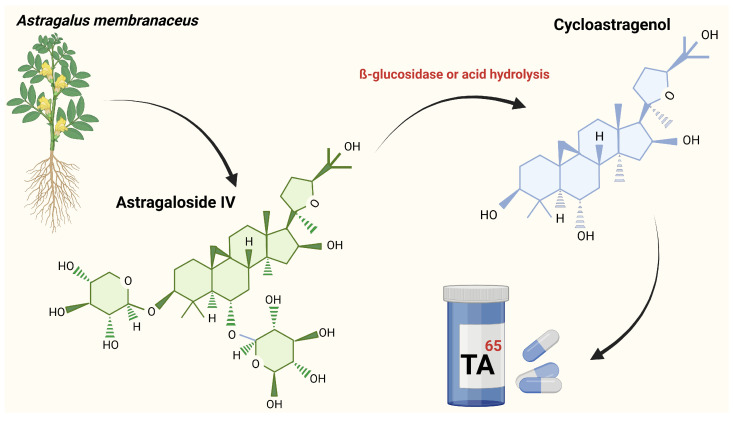
A schematic for the conversion of astragaloside IV to cycloastragenol by β-glucosidase or by acid hydrolysis. Created using https://BioRender.com.

Among these constituents, astragalosides (triterpenoid saponins) are of particular interest in terms of bioactivity. They are unique to Astragalus roots and have been named as astragaloside I, II, III, and IV [2,14], which can hydrolyze into sapogenols, like cycloastragenol. Cycloastragenol (CAG) does not exist in large amounts naturally but is derived by the hydrolysis of astragalosides, as shown in Figure 1, and has potential as a telomerase activator, which is why it serves as the basis for the commercial compound TA-65 [2].

The root of *A. membranaceus* is primarily sourced from China, Mongolia, and parts of Russia. It contains a variety of secondary metabolites including astragalosides [2]. The plant’s traditional indications, like immune enhancement, hepatoprotection, and diuretic and antihypertensive effects, are now supported by molecular studies demonstrating senescence modulation, senescence-associated secretory phenotype (SASP) suppression, and induction of apoptosis in senescent cells [2,3].

Cycloastragenol and astragalosides have demonstrated multi-pathway biological effects, including modulation of telomerase activity, antioxidative stress regulation, improved lipid metabolism, and potential anticancer properties [2,14]. These findings position *A. membranaceus* as a promising natural senotherapeutic agent with applications in skin rejuvenation, bone health, and oncology.

Other phytochemicals, such as formononetin, calycosin, and polysaccharides, contribute to the broad biological activities of *A. membranaceus*, including immunomodulation, anti-inflammatory effects, antioxidant capacity, and anti-aging actions [1]. Table 1 summarizes the phytochemicals that have a biological relevance in this species.

## 3. Telomere Elongation

Telomeres are repetitive DNA–protein structures located at the ends of chromosomes. In vertebrates, they consist of tandem repeats of the hexameric sequence (TTAGGG) that protect chromosomes from degradation and end-to-end fusion [2]. Telomerase is an RNA-dependent DNA polymerase composed of the catalytic reverse transcriptase subunit (TERT) and the telomerase RNA component (TERC). The TERC template guides the addition of telomeric repeats to chromosome ends, counteracting the gradual telomere erosion that occurs during DNA replication. The primary function of telomerase is the elongation and maintenance of telomere length [23]. Therefore, the higher expression of TERT will be reflected as higher telomerase activation and subsequently increased telomere length in certain cell types.

High levels of telomerase expression and activity are found in neural stem cells and progenitor cells; however, this activity typically declines with successive cell divisions and upon cellular differentiation or death [23]. Critically short telomeres activate DNA damage response pathways, meaning that the length of the shortest telomere, rather than the average telomere length, is the key determinant of cell viability and chromosomal stability [2].

Telomerase activators are plant-derived supplements purified from various botanical sources. Examples include salidrosides from the rhizome of *Rhodiola rosea*; TA-65 (cycloastragenol) from the roots of *Astragalus membranaceus*; DLBS1649 from *Centella asiatica*; sapogenins from *Spirulina platensis*; and *Cistanche deserticola* polysaccharides extracted from the stems [3]. The present study focuses on telomerase activators derived from *Astragalus membranaceus*, given their reported ability to stimulate telomerase activity and extend telomere length, making them promising natural nutritional supplements for supporting healthy aging.

### 3.1. Cell Culture Studies

In an in vitro study, researchers used hematopoietic progenitor stem cells and mouse embryonic fibroblasts (MEFs) from cells that are haplo-insufficient for TERC wherein the telomerase activity in these cells is reduced. These cells have only one functional copy of the RNA component of telomerase (i.e., TERC+/−). When treated with cycloastragenol, telomerase reverse transcriptase (TERT) expression increased and telomerase activity enhanced in these cells [2]. Treatment with 3 μM cycloastragenol for 6 days significantly increased telomerase activity in both rat PC12 and human SH-SY5Y neuronal cell lines. Also, an observed increase in hTERT mRNA expression and protein levels in human embryonic kidney cells (HEK-NTERT) has been reported [23]. Likewise, it was observed that cycloastragenol at various concentrations (0, 1, 2, 3, 5, and 10 μg/0.5 mL) substantially enhanced telomerase activity in primary cultured bovine cumulus cells [24].

**Figure 2:**
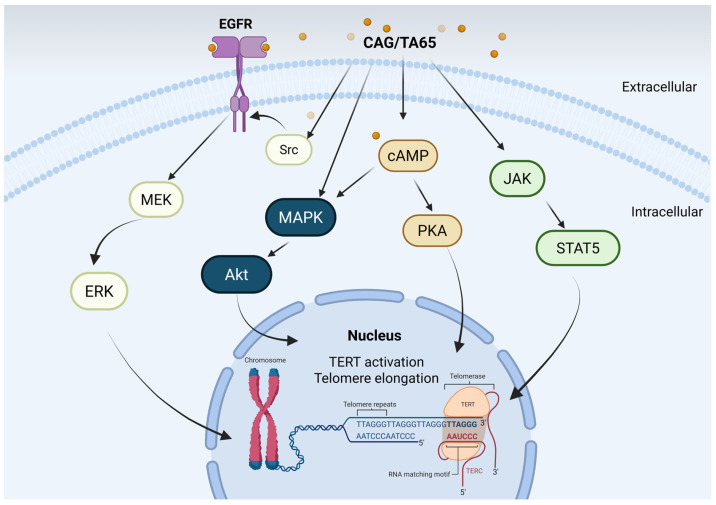
Proposed molecular mechanisms underlying telomerase reverse transcriptase (TERT) activation pathways by CAG. Cycloastragenol (CAG) or TA-65 may activate telomerase and cause cell proliferation. CAG stimulates the proto-oncogene tyrosine-protein kinase Src, which in turn activates the MEK/ERK signaling cascade; Src also activates epidermal growth factor receptor (EGFR). CAG also stimulates c-AMP, which in turn activates PKA, then phosphorylates the PKA/CREB pathway. Additionally, CAG activates Janus kinase (JAK2), which in turn phosphorylates signal transducer and activator of transcription 5 (STAT5). CAG directly and indirectly phosphorylates MAPK and activates the MAPK/Akt pathway. Ultimately, all these pathways activate TERT. These proposed mechanisms are based on currently available experimental evidence [2,3]. Created using https://BioRender.com.

Several studies have investigated the cycloastragenol/TA-65 cascade. Cycloastragenol has been reported to enhance cell proliferation, presumably through the modulation of the Src/MEK/ERK signaling pathway [2,23], leading to the phosphorylation of extracellular signal-regulated kinase (ERK) in many cell lines such as HEK293, and HE-neo in keratinocytes, lung, brain, endothelial, and hematopoietic stem cells (HSCs). Furthermore, the proto-oncogene tyrosine-protein kinase (c-Src), ERK kinase, and epidermal growth factor receptors are involved in the cycloastragenol-induced ERK phosphorylation pathway (Figure 2). It also potentially acts through the involvement of the JAK/STAT signaling pathway, where cycloastragenol induces Janus kinase 2 (JAK2) expression, then activates STAT5 by phosphorylation, leading to increased TERT expression [2]. Cycloastragenol activation of telomerase is also linked to the cAMP response element-binding protein (CREB), a protein that regulates gene expression [2]. Astragaloside has also been associated with increased telomerase activity, likely through modulating MAPK and Akt signaling pathways, leading to the phosphorylation of TERT through post-transcriptional modification, thus promoting telomerase activity [3].

In summary, the available in vitro evidence consistently demonstrates that cycloastragenol may enhance telomerase activity across multiple cell types by upregulating TERT expression and activating key signaling pathways. Mechanistically, its effects are presumably mediated through pathways such as Src/MEK/ERK, JAK/STAT, MAPK, Akt, and CREB, all of which contribute to telomerase activation and cellular proliferation. These findings suggest that cycloastragenol holds potential as a modulator of telomerase function; however, further studies are needed to confirm its efficacy and safety in vivo and to better understand its broader biological implications.

### 3.2. Animal Studies

In an in vivo study, 1- and 2-year-old female mice were given dietary supplementation with TA-65 (25 mg of TA-65 per kg of mouse body weight per day, mixed in 100 μL of fruit mash) for 4 months. It was found that telomerase reverse transcriptase (mTERT) mRNA expression and protein levels in mouse liver were significantly increased by about 10-fold, but there were only small to modest increases in mTERT mRNA in other tissues such as the kidney, lung, and brain [25]. Moreover, the critically short telomeres percentage was reduced in haplo-insufficient mice embryonic fibroblasts that harbor critically short telomeres. The authors concluded that TA-65 did not significantly extend the overall lifespan in treated vs. control female mice, but led to an enhancement of the overall health-span indicators such as glucose tolerance, osteoporosis, and skin fitness [2,25]. This differential response of different tissues’ telomeres to TA-65 was also reported by Wolf et al. (2021) who showed that 8 days of oral TA-65 (0.1, 0.2 mg per g body weight) administration to swallow chicks (starting at 4 days after hatching) resulted in telomere lengthening in blood cells and accelerated feather growth, without changes to chicks’ mass over time. It was surprising that TA-65 had no effect on telomere length in the brain or spleen; it even caused telomere shortening in the liver and adrenal gland [26]. This experiment revealed limitations to telomere protection in biological senescence, for it revealed that telomere dynamics in the life of developing juvenile birds may have the potential to put the individual organism on its trail of senescence affecting future health- and lifespan outcomes.

In summary, in vivo studies suggest that TA-65 can enhance telomerase activity and improve certain health-span indicators, despite not significantly extending overall lifespan. Its effects appear to be tissue-specific, with notable increases in telomerase expression in some organs but limited or even adverse effects in others. These findings highlight the complexity of telomere regulation in living systems and underscore the need for further research to clarify the long-term implications and variability of TA-65 across different tissues and developmental stages.

### 3.3. Human and Clinical Studies

There are limited studies on humans in which Astragalus extract or its compounds or preparations has been tried. For example, the effects of hydroethanolic root extract of *A. mongholicus* (containing 0.05% formononetin and 0.16% of astragaloside IV, isoflavones, including calycosin, ononin, and calycosin-7-O-d-glucoside) and danazol, a synthetic steroid drug used as a positive control, have been examined on lymphocytes of people of ages ranging between 2 and 76 years old, where telomeres shorten by about 79 base pairs/year. It was found that 13 out of 18 donors (72.2%) who took the root extract showed telomere lengthening, whereas only 9 out of 18 donors (50%) showed telomere lengthening after danazol treatment, and cells with very short telomeres decreased in all donors in both groups. Both treatments increased hTERT compared to untreated lymphocytes, but danazol was more powerful than the root extract [15].

In another randomized, double-blind, placebo-controlled study, TA-65 was administered to 117 relatively healthy adults (age: 53–87 years) and the telomere length of their peripheral blood mononuclear cells (PBMCs) was measured. It was found that a low dose of TA-65 (250 U) can significantly (*p* = 0.005) increase telomeres’ lengths by 530 ± 180 bp over 12 months, whereas the placebo group significantly lost telomere length (290 ± 100 bp, *p* = 0.01). A high dose of TA-65 (1000 U) caused an insignificant trend of increase in telomere length compared to that of the placebo group. These authors concluded that TA-65 can lengthen telomere length in a possibly clinically significant manner [27].

In a third randomized, double-blind, placebo-controlled clinical trial on patients with metabolic syndrome, the consumption of 16 mg of TA-65 daily for 12 weeks or a placebo indicated a significant effect for the telomerase activator [28]. For example, risk factors for cardiovascular disease were improved; there was a reduced atherosclerotic index (LDL-c/HDL), reduced inflammatory markers like TNF-α, reduced body mass index, and reduced waist circumference [29,30]. These enhancements have been attributed to telomerase activation induced by TA-65.

While both clinical and preclinical studies suggest that *A. membranaceus* and its derivative TA-65 may enhance telomerase activity and support telomere maintenance, much of the available evidence is derived from in vitro and animal studies. Clinical data on humans remain limited and, in some cases, inconsistent, and the overall effects on telomere dynamics are not yet definitively established.

## 4. Anti-Aging Effects

Aging is characterized by a progressive decrease in intrinsic physiological function and an increased incidence of chronic diseases, which lead to an increase in age-specific mortality rate and decrease in age-specific reproductive rate [31,32]. Cellular senescence, a state of permanent cell cycle termination, is one of the main hallmarks of aging, and the main feature of senescent organs is the accumulation of senescent cells [33]. Several hallmarks such as genomic instability, telomere attrition, epigenetic alterations, decline in proteolysis, mitochondrial dysfunction, cellular senescence, stem cell exhaustion, and altered intercellular communication have been described for aging cells. These changes not only lead to the acceleration of the progression of aging but also to the development of age-related chronic diseases such as cardiovascular diseases, diabetes, cancers, neurodegenerative pathologies, and neurodegenerative diseases [32,33]. Thus, it is imperative to develop more efficient and cost-effective anti-aging or healthy aging therapies [34].

### 4.1. Aging of the Bone (Osteopenia and Osteoporosis)

Bone loss is the result of an imbalance between osteoclasts and osteoblasts functions. The most prevalent bone diseases are osteopenia and osteoporosis [35]. Osteoporosis, often referred to as the “silent bone thief”, is a condition marked by decreased osteoblast activity, leading to reduced bone density, weakening, and fragility. In men, osteoporosis typically manifests after the age of 70, whereas in women, osteoporosis is diagnosed earlier, typically after the menopause [35]. Senile osteoporosis, a common skeletal disease closely associated with low quality of life among the aged population, is characterized by decreased osteoblast activity, low bone mass, and microarchitectural deterioration of bone tissue, bone microstructure, and increased bone brittleness [36]. Several studies with *A. membranaceus* or its products have been performed in in vitro, in vivo, and clinical settings.

#### 4.1.1. Studies at the Cellular Level

**Figure 3:**
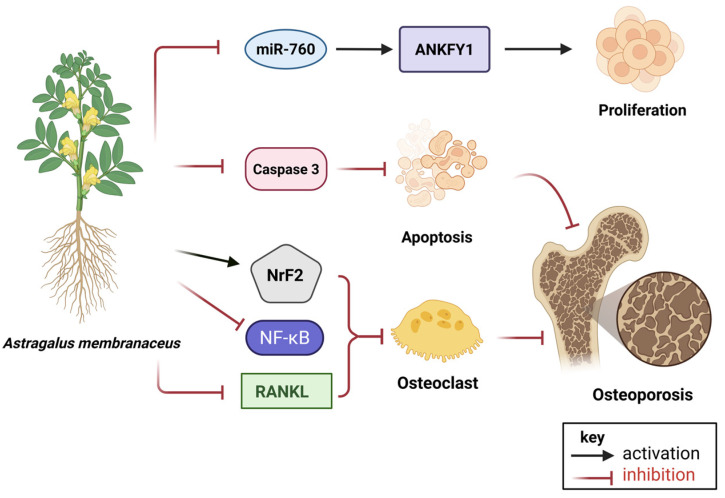
Proposed cellular mechanisms underlying the effects of *A. membranaceus* on bone aging. *A. membranaceus* inhibits osteoclasts by suppressing the NF-κB pathway, disrupting Keap1-Nrf2 association to enhance antioxidant defense, and downregulates RANKL. Osteoporosis is prevented by the deactivation of the caspase pathway and cell proliferation is enhanced through the inhibition of miR-760 and increased ANKFY1. These proposed mechanisms are based on currently available experimental evidence [37,38,39,40]. Created using https://BioRender.com.

Several studies have been done at the cellular level. In a pilot study, rat primary nucleus pulposus cells extracted from the center of rat intervertebral discs showed increased expression levels of both pro-apoptotic proteins, cleaved caspase-3, and Bcl-2-associated X protein (BAX), as a result of glucose-induced stress, whereas anti-apoptotic protein B-cell lymphoma 2 (Bcl-2) expression significantly decreased. Cycloastragenol or astragaloside (AG-IV) treatment at concentrations of 1, 3, and 5 µM reduced cleaved caspase-3, decreased the expression of BAX, and increased the expression of Bcl-2 as compared to high glucose alone [14]. Researchers studied the role of miR-760 in osteoporosis and its effect on human bone marrow stem cells (h-BMSCs) treated with Astragalus polysaccharides (APSs). miR-760 was found to be higher in osteoporosis, whereas 200 µg/mL APS lowered miR-760 in h-BMSCs by about 48%. This inhibition of miR-760 increased cell viability of the h-BMSCs cell line to 150% and elevated the expression levels of the osteogenic marker genes RUNX2, ALP, and OCN mRNA levels, which were upregulated. The inhibition of miR-760 in h-BMSCs led to increased ANKFY1 expression by ~2.89-fold, and this increase in ANKFY1 was associated with enhanced cell proliferation and higher levels of cyclin D1, a key protein that controls cell cycle progression [37]. APS treatment significantly inhibited the receptor activator of nuclear factor-κB ligand (RANKL) that is known to induce osteoclast differentiation in vitro. When Han et al. (2021) [38] cultured bone marrow mononuclear cells with RANKL alone, a substantial number of osteoclast-like cells formed. In contrast, cultures treated with APS, particularly at 100 ng/mL, showed a marked reduction in osteoclast-like cell formation. Furthermore, APS treatment led to significantly decreased mRNA expression levels of tartrate-resistant acid phosphatase (TARP), receptor activator of nuclear factor-κB (RANK), and TNF receptor-associated factor 6 (TRAF6)—all of which are key genes involved in RANKL-mediated osteoclastogenesis—compared to the RANKL-only-treated group. The authors reported that when RANK binds to RANKL, its intracellular part is activated, then combined with TRAF6, causing the phosphorylation of κB inhibitory kinases, thus activating NF-κB, which causes TARP expression and maturation of osteoclasts. APS has been found to inhibit this pathway. These findings indicate that APS exerts inhibitory effects on osteoclast differentiation through the downregulation of RANKL-associated pathways [38]. Wang et al. (2022) [39] demonstrated that CAG inhibited RANKL-induced osteoclast differentiation in the RAW264.7 cell line by preventing IκBα degradation, which in turn suppressed NF-κB nuclear translocation, and suppressed Ca^2+^ oscillations and the subsequent activation of the nuclear factor of the activated T cell 1 (NFATc1) pathway. CAG also inhibited osteoclast differentiation by enhancing the antioxidant defense system of the cell by causing nuclear factor erythroid 2-related factor 2 (Nrf2) nuclear translocation [39]. Similarly, bone marrow mononuclear cells (BMMCs) isolated from the femurs and tibias of mice were induced to differentiate into osteoclasts using RANKL and macrophage colony stimulating factor (M-CSF). CAG treatment markedly reduced osteoclast formation and the expression of osteoclast-specific markers in BMMC cultures by inhibiting NF-κB and the calcium/nuclear factor of activated T cell 1 (Ca^2+^/NFATc1) signaling while activating the Nrf2/Kelchlike ECH-associated protein 1/antioxidative response element (Nrf2/Keap1/ARE) pathway that scavenges reactive oxygen species (Figure 3) [39]. Yu et al. (2020) [40] also used osteoblastic MC3T3-E1 cells, which closely resemble primary calvarial osteoblasts, to assess osteogenic activity. Treatment with CAG significantly increased cell viability, caused osteoblastic differentiation, and mineralization in a dose-dependent manner (0.03–0.3 μM). These findings indicate that CAG effectively promotes osteogenic activity and has strong potential to enhance bone formation [40].

These studies indicate that CAG, AG-IV, and APS exert their effects through the inhibition of miR-760, which leads to increased ANKFY1, and therefore to the proliferation of bone. Osteoporosis can be inhibited through the deactivation of caspase-3, and osteoclast differentiation is inhibited through the nuclear translocation of the antioxidant defense system of Nrf2, the suppression of NF-κB, and the inhibition of RANK and its ligand.

#### 4.1.2. In Vivo Studies

Yu et al. (2020) [40] used 2 models of aging to study the effects of CAG: D-galactose-induced aging given to 59-week-old rats for 20 weeks and natural aging of 51-week-old rats. The animals were administered CAG for 20 and 33 weeks. The authors found that the D-galactose model is better for testing the effect of compounds on bone formation and resorption, although this model does not fully represent bone loss. The study found that CAG potentially prevents bone loss by increasing the expression of osteoactivin, a transmembrane glycoprotein that is vital for bone formation [40].

Cycloastragenol treatment has been found to effectively mitigate bone resorption and oxidative stress in both D-galactose-induced and naturally aged rats. It significantly reduced serum tartrate-resistant acid phosphatase levels (TRACP), a biomarker for bone resorption and inflammation, and oxidative stress markers like malondialdehyde (MDA). Cycloastragenol also improved redox balance; enhanced bone mechanical properties; increased rigidity, maximum load, and elastic load; preserved trabecular microarchitecture; attenuated the loss of trabecular number and thickness; reduced separation; and increased bone mineral density in addition to promoting bone formation. This treatment also reduced osteoclast numbers, upregulated osteoactivin, supported osteogenic differentiation, and restored bone remodeling balance [40].

Other works have studied the effect of *Astragalus* on laboratory animals. Old female mice were treated with TA-65, and bone mineral density was compared to the untreated group. Bone mineral density was increased and this improvement translated into stronger, healthier bones and reduced fragility risk in aged female mice treated with TA-65. Notably, the treatment did not cause abnormal bone growth, tumors, or skeletal deformities. Its effect was restorative rather than excessive [25]. Han et al. (2021) [38] used cotton ligature to induce periodontitis in rats and demonstrated that Astragalus polysaccharide (APS) attenuates alveolar bone resorption in periodontitis by modulating the local osteoimmuno-cytokine network. Specifically, APS suppressed RANKL expression and partially restored the balance of anti-inflammatory cytokines such as IL-10 and TGF-β, thereby contributing to bone homeostasis and mitigating inflammatory responses in periodontal lesions through immunoregulatory mechanisms. The study found that APS treatment significantly modulated the local cytokine environment associated with bone resorption in periodontitis. mRNA expression of RANKL was markedly elevated in the gingiva of untreated periodontitis rats compared to healthy controls, whereas APS-treated rats showed a significant reduction in RANKL levels relative to the experimental periodontitis group. In contrast, the expression of osteoprotegerin, a decoy receptor that inhibits RANKL activity, remained largely unchanged among groups, except in the healthy group, which exhibited slightly lower levels. Regarding the anti-inflammatory cytokines, both IL-10 and TGF-β mRNA levels were significantly higher in healthy rats than in untreated periodontitis rats. APS-treated rats showed a modest increase in these cytokines, with TGF-β expression remaining significantly lower than that in the healthy group [38].

In another study, mice with osteoporosis (SAMP6, a strain of accelerated senescence) had osteoporosis that worsened with time; trabeculae were thinner, broken, and uneven; reticular bone structure was destroyed; and cortical bone was much thinner, with severe bone loss and a damaged bone network. *A. membranaceus* root extract ameliorated these symptoms including bone loss, which was much milder in treated mice than in the control group; mice had thicker trabeculae, compact and evenly arranged bone, nearly complete reticular (mesh-like) structure, although the cortical bone was still somewhat thinner. This improvement was attributed to the contents of calcium and phosphorus in the femur of SAMP6 mice being increased by *A. membranaceus* root extract. It was observed that the expression of mRNA and protein of FGF23 and CYP24A1 (promotes vitamin D decomposition) levels decreased in bone marrow stem cells and blood while Klotho (a longevity protein), vitamin D receptor, CYP27B1 (provides instructions for making the enzyme 1-alpha-hydroxylase, thus promoting vitamin D synthesis), and bone Gla protein levels increased in bone marrow stem cells and blood, thus preventing osteoporosis [36].

*A. membranaceus* extract inhibited ovariectomy-induced bone mineral density reduction in the L4 vertebrae and femurs in rats, reduced deoxypyridinoline and bone resorption marker levels, and prevented the deterioration of trabecular microarchitecture, which was accompanied by a significant decrease in skeletal remodeling [41]. Osteoporosis, characterized by reduced bone mass and microstructure damage, is largely driven by excessive osteoclast activity and bone resorption, a condition associated with aging. Kang et al. (2013) demonstrated that ovariectomized mice treated with CAG exhibited reduced bone loss and improved bone microarchitecture, showing denser and more connected trabecular bone with a significantly higher bone volume/tissue volume, trabecular number, and connectivity density as compared to untreated ovariectomized mice [42].

In summary, multiple in vivo studies demonstrate that cycloastragenol (CAG), TA-65, and Astragalus-derived compounds exert protective effects against age-related bone loss. These agents improve bone mineral density, enhance bone strength and microarchitecture, and restore the balance between bone formation and resorption. Mechanistically, their benefits are linked to reduced oxidative stress and inflammation, suppression of osteoclast activity, and modulation of key signaling molecules involved in bone remodeling. Overall, these findings suggest that Astragalus-derived compounds hold promise as therapeutic agents for preventing or mitigating osteoporosis and other bone-related aging conditions.

#### 4.1.3. Clinical Studies

In women undergoing menopausal transition-related symptoms, the administration of Astragalus extract over three months has been shown to stimulate estrogen secretion and to prevent the onset of osteoporosis by increasing the proliferation of osteoblasts [35]. Noteworthy, calycosin, an isoflavonoid phytoestrogen extracted from the root of the medicinal herb *A. membranaceus* (Table 1), prevented osteoporosis in postmenopausal women by stimulating osteoblast differentiation due to modulating the GSK-3β pathway and increasing the levels of specific markers of osteoblast differentiation such as alkaline phosphatase, alpha-1 type I collagen, and Runx2 protein. Furthermore, calycosin has been found to stimulate the expression of osteoprotegerin, which is an osteoclastogenesis inhibitory factor. Correlated with the MAPK pathway, calycosin was able to abolish RANKL-induced osteoclast formation from primary bone marrow macrophages [35]. Another clinical observation was reported in a study that included 26 subjects who took 5–50 mg of TA-65 daily for a year. It was noted that TA-65 supplementation led to a statistically significant increase in bone mineral density at the lumbar spine (L1–L4), which is a common site of osteoporotic fractures, lowered osteoclast activity, and slowed age-related bone loss where no significant change was observed in the hip [43].

The findings of the overall studies suggested that *Astragalus* has an anti-postmenopausal osteoporotic effect by mitigating bone fragility and chronic pain, improving bone microstructure, protecting bone against skeletal deterioration, enhancing osteoblast activity, and reducing osteoclast numbers and their mobility. Concurrently, the reduction in bone aging is associated with increased activity of the aging suppressor gene (alpha Klotho protein) and increased mineral (calcium and phosphate) concentration within the bone system (Table 2).

### 4.2. Aging of the Skin

Skin aging is a natural biological process that occurs over time and affects most cells, organisms, and species, and in humans its visible effects can be a cause of concern for many individuals [3,46]. The aging process is influenced by a number of internal and external agents, including environmental factors such as ultraviolet radiation, sun exposure, and air pollution, and a poor diet that lacks nutritional value and includes smoking [35,46]. As humans age, a series of bodily changes will occur, including the degradation of subcutaneous tissue, muscles, and bones, as well as alterations in the histological and morphological structure of the skin. Notably, these changes include the atrophy of the fatty layer beneath the epidermis, a reduction in the number of fibroblasts, and a decline in the levels of intercellular matrix components, such as collagen, ceramides, and hyaluronic acid, leading to wrinkles, loss of skin elasticity, a rough-textured appearance, laxity, and sagging skin [35,48]. In recent years, there has been an observable shift towards incorporating natural plant-based ingredients in skincare products. This growing trend has prompted the development of new formulations that meet the increasing consumer demand for effective and natural plant-based skin rejuvenation solutions [48].

**Figure 4:**
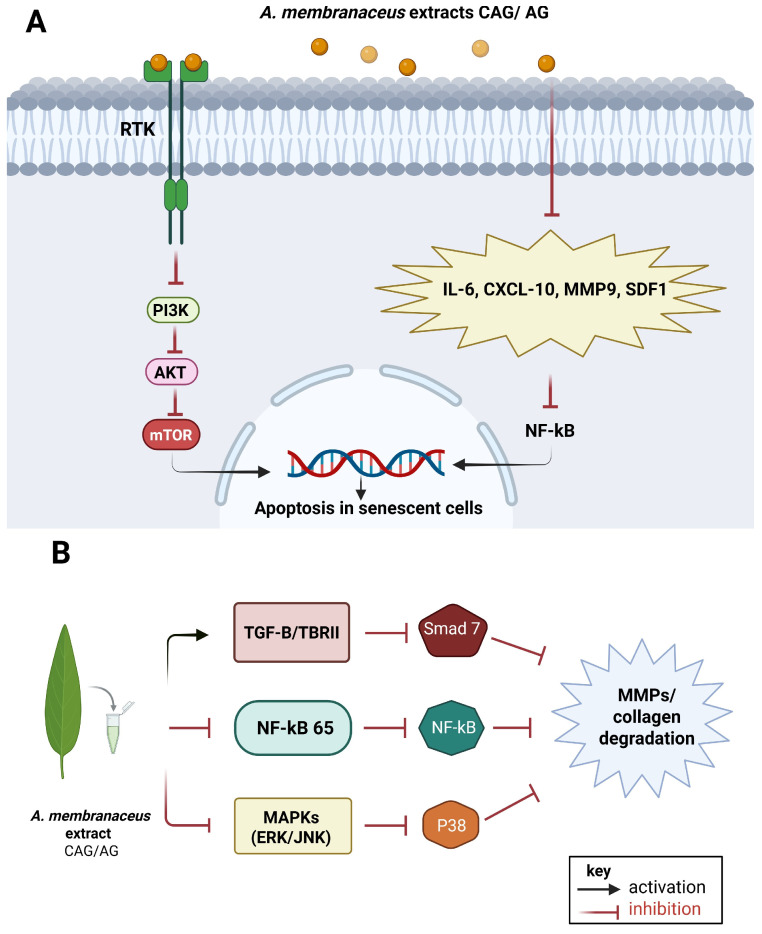
Proposed cellular pathways mediated by *Astragalus membranaceus* in skin aging. (**A**) *A. membranaceus* extracts induced apoptosis of senescent cells by reducing senescence associated factors (IL-6, CXCL-10, MMP9, SDF1), leading to the inhibition of NF-κB. CAG treatment also caused the inhibition of the phosphorylation of PI3K, AKT, and mTOR. Binding of *A. membranaceus* with RTK receptor in senescent cells triggers apoptosis through the inhibition of the anti-apoptotic Bcl-2 family and subsequently the PI3K/AKT/mTOR pathway. (**B**) *A. membranaceus* extracts inhibited the activation of matrix metalloproteinases (MMPs) by many pathways; *A. membranaceus* suppressed the NF-κB P65 pathway, it also inhibited the mitogen-activated protein kinase (MAPK) signaling cascade involving ERK, JNK, and p38 proteins. The extracts also maintained the integrity of the TGF-β pathway but downregulated SMAD7. The downregulation of MMP reduced the degradation of skin collagen. These proposed mechanisms are based on currently available experimental evidence [45,47,48,49]. Created using https://BioRender.com.

In humans, IMR-90 fibroblast non-senescent cells and etoposide-induced senescent cells (VP16-SCs) were treated with 100 μM of cycloastragenol. The treatment reduced the viability of senescent cells but had no toxicity on non-senescent cells regardless of whether senescence was induced by etoposide (VP16-SCs) or by repetitive replication. CAG also lowered mRNA and protein levels of major senescence-associated secretory phenotype factors like IL-6, CXCL-10, MMP9, and SDF1 in senescent IMR-90 fibroblasts and human embryonic lung fibroblasts by inhibiting NF-κB and STAT3. Cycloastragenol also triggered apoptosis in senescent cells through the inhibition of the anti-apoptotic Bcl-2 family and PI3K/AKT/mTOR pathway (Figure 4A). In addition, CAG suppressed senescence-associated secretory phenotypes (SASPs), and therefore inhibited migration induced by SASPs [32]. In two separate experiments, matrix metalloproteinases (MMPs) activated by UV radiation led to collagen degradation by collagenase in human foreskin fibroblasts (HS68 cell line) and human dermal fibroblasts (HDFs). *A. membranaceus* extracts inhibited the NF-κB P65 (MAPKs: ERK, JNK, p38) pathway, which inhibited MMP activation, thus reducing skin collagen degradation, and in the HS68 cell line increased procollagen synthesis (Figure 4B) [45,47].

Astragaloside IV promoted collagen synthesis in aged skin fibroblasts and reduced the apoptosis of these cells [3]. A cream containing a combination of astragaloside IV and hylasom EG10 (chemically cross-linked hyaluronic acid to enhance the penetrability of astragaloside) was tested on two cohorts of 15 and 20 participants, and their skin parameters at baseline and after 4 weeks for the first cohort, and 4 and 8 weeks of treatment for the second cohort, were measured in vivo. The cream significantly improved skin hydration by 95%, skin appeared brighter by 90%, and wrinkle visibility was reduced by 70%, and this effect was attributed to the cream’s telomere-lengthening ability [48]. Astragaloside increased the cell viability in human skin fibroblasts and protected the cells and improved their survival after UVA exposure. Ultraviolet (UV) radiation reduces type I collagen, which is the primary structural protein in the skin, by inhibiting its synthesis. Astragaloside was found to counteract this effect by preserving type I collagen levels. It achieved this by inhibiting UVA-induced MMP-1 expression and maintaining the integrity of the TGF-β/Smad signaling pathway, which is essential for collagen production. Specifically, astragaloside reduced the expression of the negative feedback regulator Smad7 and suppressed MMP-1 expression, thereby preventing the UVA-induced downregulation of TGF-βRII (Figure 4B). The skin-protecting properties of the combination cream were attributed to the ability of astragaloside to stimulate collagen production [49].

It is worth mentioning that 30% ethanol extraction of *A. membranaceus* and *Rubus coreanus* for 4 h and the lyophilization of the extract demonstrated that tablets of 1000 mg of this extract, given twice daily, demonstrated estrogenic activity in women with menopausal symptoms. This estrogen-like effect may activate estrogen receptors (particularly β receptor), which can be beneficial in maintaining skin elasticity and inhibiting collagen breakdown [35]. This potential estrogenic activity may interfere with treatments that are intended to suppress estrogen-like breast cancer.

Liu et al. (2017) reported that calycosin suppressed melanin formation, contributing to improved skin appearance and protection against photoaging [31]. The effect of saponins from *A. membranaceus* and *Centella asiatica* dried extract was tested on 150 healthy volunteers who received either topical extract cream, oral extract capsules, combined treatments, or corresponding placebos. The end-point measurement showed that the combined oral and topical application of creams prepared from the extract yielded the most comprehensive and significant benefits, such as improving skin hydration, brightness, firmness, pigmentation balance, pore appearance, texture, and collagen content [46].

TA-65 was found to improve signs of premature skin aging. After eight weeks of daily capsule administration, volunteers showed no signs of significant skin damage, uneven skin pigmentation, or wrinkles. Histological analyses of biopsies revealed that daily intake of TA-65 increased elastin levels after sixteen weeks [28]. In another study, 20 healthy women aged 40–65 were divided into groups using either an active regeneration booster cream containing cycloastragenol as a key ingredient (AM/PM) or a placebo for 12 weeks. The results showed significant improvements in skin texture and reductions in fine lines on the cheeks and around the eyes among those using the active formula. All participants who received the cycloastragenol cream reported visible improvements across multiple skin categories, with 100% showing enhancement in at least three aspects of skin appearance. No sensitivity or irritation was reported [46]. The results of this study, however, are complicated by the fact that the cream used contained many skin-protecting compounds such as TGF-β-1 (rh-polypeptide-22), epidermal growth factor (sh-oligopeptide-1), keratinocyte growth factor (sh-polypeptide-3), peptides (palmitoyl pentapeptide-4, myristoyl pentapeptide-8, myristoyl pentapeptide-11, and myristoyl tetrapeptide-12), green tea extract, coenzyme Q10, hyaluronic acid, and essential fatty acids, in addition to cycloastragenol.

In animal model experiments, mice were exposed to a sublethal dose of total-body irradiation for two months, where they aged with greyed fur and weak bones. Mice were then treated with 50 mg/Kg cycloastragenol daily for 2 weeks by oral gavage. Cycloastragenol improved greyed fur, rickets, and restored bone mineral density (431.6 mg/cm^3^ vs. 394.5 mg/cm^3^) in the irradiated group, as well as increasing stem cell numbers [32].

In summary, *A. membranaceus* contains compounds like cycloastragenol and astragaloside IV that have demonstrated potential skin-protective effects, including improvements in skin elasticity and thickness, reduced wrinkle appearance, protection against UV-induced damage and photoaging, modulation of melanogenesis, and support of collagen production. These effects may involve the regulation of inflammatory pathways, including TNF-α signaling, as well as influences on telomere maintenance and cellular stress responses. Most of these skin-enhancing properties have been observed in animal models, with limited evidence also available from human studies (Table 2).

## 5. Anticancer Effect

Cancer is a major public health problem characterized by a group of illnesses that can impact any area of the body, with rapid proliferating aberrant cells that invade neighboring body parts and spread to other organs [50]. Cancer is a leading cause of death globally; according to the data from the World Health Organization, cancer is the first or second leading cause of death in 183 countries and third or fourth in other regions [51]. Cancers develop due to genetic alterations as well as the accumulation of epigenetic alterations (DNA methylation, dysregulation of microRNAs, and histone modification) [52]. Cancer treatment strategies include surgical excision, chemotherapy, radiotherapy, hormone therapy, and targeted therapy. However, based on clinical factors related to treatment, these options can lead to many side effects, such as postoperative tumor spread, metastasis, or resistance to chemotherapy, and therefore significantly impact a patient’s prognosis [51].

The correlation of cancer to telomere length is rather complex. It has long been assumed that telomerase activation and, consequently, lengthy telomeres, are associated with cancer development and immortalized state. This assumption stemmed from the observations that many viral infections such as Epstein–Barr virus or cytomegalovirus are associated with malignancies that encode for viral proteins that indirectly (through proto-oncogenes) turn on telomerase activity. On the other hand, many cancers are characterized by extremely short telomeres that help hide cells from immune surveillance and attack. These short telomeres, however, create instability and a crisis that may drive cancer development to which the cells respond by reactivating telomerase to maintain length and immortal growth [53]. Burke et al. (2021) reviewed the role of telomerase in aging and skin carcinogenesis, highlighted the dual role of telomerase in both normal aging (shortening) and cancer (activation), and proposed that activating telomerase early in life might prevent the instability that leads to cancer, challenging the idea that telomerase activators inherently cause tumors [54]. In the following sections, we discuss the effects of *A. membranaceus* on some bone and skin cancers and the possible mechanisms and pathways through which cancer develops.

### 5.1. Bone Cancer (Sarcoma)

**Figure 5:**
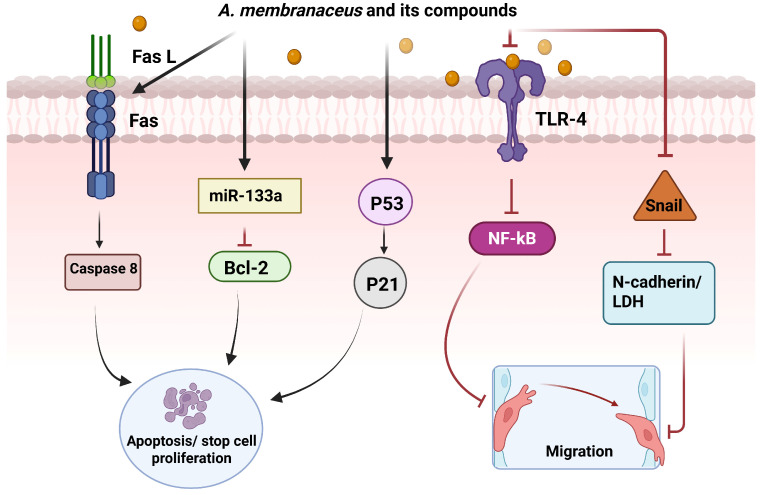
Depicted effect of *Astragalus* ingredients on tumor cell apoptosis and cell migration. *A. membranaceus* and its compounds induced apoptosis in cancer cells by different pathways; it enhanced the expression of death receptor Fas and its ligand FasL to trigger caspase pathway, and regulated miR-133a that lowers Bcl-2 that ultimately leads to the apoptosis of cancer cells. *A. membranaceus* also activated p53 leading to the upregulation of its downstream targets, including p21 and Bax, which in turn trigger cell cycle arrest and apoptosis. *Astragalus* can also trigger an anticancer cascade by preventing the migration of cancer cells through suppressing the TLR4 receptor to inhibit adaptor protein leading to the activation and phosphorylation of NF-κB, thus inhibiting tumor migration. *A. membranaceus* also prevents tumor cell migration through the inhibition of the snail cascade. These proposed mechanisms are based on currently available experimental and associative evidence [51,58]. The head arrow indicates activation, while the blunt-ended red arrow indicates inhibition. Created using https://BioRender.com.

In animal models, osteosarcoma xenograft mice have been used to clarify the effect of astragaloside IV. This compound reduced tumor growth by the activation of the death receptor pathway (Fas/FasL, caspas-8, caspase-3) and mitochondrial apoptosis (Figure 5) [51]. *Astragalus* (Huangqi) injection, which contains CAG as an important ingredient, significantly suppressed osteosarcoma growth in vivo by enhancing cellular immunity through increasing the number of CD8^+^ T cells in mice implanted with osteosarcoma tumors, without causing significant toxic side effects. Molecular docking showed that CAG activates cytotoxic T lymphocytes and inhibits cathepsin L expression, and therefore has antiosteosarcoma effects [55]. S180 sarcoma tumor-bearing mice have been used to evaluate the antitumor effects of *A. membranaceus* polysaccharide. Astragalus polysaccharides effectively regulated the percentages of CD3+, CD4+, CD8+ T cells, and CD19+ B cells in S180 tumor-bearing mice in a dose-dependent manner, and inhibited the growth of solid tumors by increasing anaerobic metabolism in the tumor cells leading to tumor cell apoptosis [56].

In the human skeletal system, osteosarcoma is becoming the second leading cause of cancer-related deaths in children and adolescents worldwide [57]. Astragaloside (AS-IV) inhibited cell proliferation and promoted apoptosis in the osteosarcoma 143B cells line, through the upregulation of the death receptor Fas/FasL-caspase signaling pathway [51,57]. It seems to serve as a chemosensitizer that boosts chemotherapy effectiveness through Fas/FasL signalling in human osteosarcoma cell lines MG-63 and 143B, and the BALB/c nu/nu mice xenograft. Human osteosarcoma cells (MG-63) have been cultured with Astragalus polysaccharides as treatment at different concentrations. These polysaccharides significantly reduced the proliferation of MG-63 osteosarcoma cells in a dose-dependent manner, suppressed their migration and invasion, and promoted apoptosis by downregulating anti-apoptotic Bcl-2, while upregulating Bax, cleaved caspase-3 (pro-apoptotic), and microRNA-133a, which led to the decreased expression of JNK proteins, and therefore the MAPK pathway [58]. These authors noted that APS also caused cell cycle arrest in the S phase and apoptosis, and the anti-migratory effect was reflected by lower levels of MMP-2 and MMP-9.

In summary, Astragalus-derived compounds, including astragaloside IV, cycloastragenol, and Astragalus polysaccharides, demonstrate significant anti-osteosarcoma activity in both in vivo and in vitro models. Their effects are mediated through the induction of apoptosis via death receptor and mitochondrial pathways, inhibition of tumor proliferation, and suppression of migration and invasion. Additionally, these compounds enhance immune responses by modulating T- and B-cell populations and may improve chemotherapy efficacy. Overall, these findings highlight the potential of these compounds as promising adjuncts in osteosarcoma treatment.

### 5.2. Skin Cancer

Skin is a layer that protects our body from various environmental damages. It is constantly exposed to external and internal stressors, which further threaten its genomic integrity, potentially causing unwanted changes at the molecular and cellular levels [50]. Skin cancer is one of the most frequently occurring types of cancer and accounts for most malignancies across the globe [59]. It is generally classified into melanoma and non-melanoma skin malignancies [51]. Malignant melanoma, originating from the dysplasia of melanocytes, is characterized by increasing incidence and high metastatic capacity [60]. Surgery is the primary treatment for malignant skin cancer in its early stages [61], while chemotherapy and radiotherapy are employed in patients unsuitable for surgery and those refusing surgery, but they are prone to side effects and poor prognosis. Therefore, there is an urgent need to explore new treatment methods for malignant skin cancer. Moreover, the potential values of traditional Chinese medicine in the treatment of melanoma have gradually been revealed [60].

Melanoma cell line A375 cells were treated with TA-65 at different concentrations (150, 200, and 250 µg). This caused a reduction in cell proliferation and viability after 48 h of treatment. TA-65 induced the death of cancer cells through upregulating the expression of histone deacetylase 8 (HDAC8) and HDAC10. On the other hand, hTERT gene expression was downregulated in treated groups; thus, TA-56 might act as a potential epigenetic regulator in melanoma [52]. Human malignant melanoma A375 cell proliferation was significantly inhibited by a mixture of *A. membranaceus* and *Radix trichosanthis* decoctions (prepared by soaking 100 g of plant material for 1 h, then concentrated to 50 mL and centrifuged at 1000 g, when the supernatant was used) in a dose-dependent manner. It was found that the group treated with the combination exhibited better protective effects against proliferation than the single decoction of each plant; the decoction combination promoted A375 cell apoptosis by reducing Bcl-2 expression and enhancing the Bax protein level [60]. Furthermore, the mixture of *A. membranaceus* and *R. trichosanthis* inhibited A375 cell migration by increasing E-cadherin, a protein that helps epithelial cells stick tightly to each other, maintaining tissue integrity and preventing cells from moving around too much. They also decreased N-cadherin, a protein more common in mesenchymal cells; decreased snail cascade and LDH activity, the key enzyme promoting tumor glycolysis and associated with tumor proliferation and metastasis (Figure 5); and finally reduced the expression of Akt through the inhibition of Akt phosphorylation of A375 cells [60].

In the vulvar squamous carcinoma SW962 cell line, astragaloside inhibited cell proliferation and promoted autophagy by regulating lysosomal enzymes and autophagy-related proteins through the downregulation of many cancer-associated genes/factors such as toll-like receptor 4 (TLR4), transforming growth factor beta (TGF-β), and MMP-9. On the other hand, tumor suppressors such as (P53, P21, cyclin D1, Bax, and cleaved-caspase-3) were upregulated, while cancer cell migration was inhibited [51] (Figure 5).

In an in vivo experiment, male and female C57BL/6J mice aged 6–8 weeks were injected with B16-F10 melanoma cells. One group was treated with cyclophosphamide as a positive control, and the other group was treated with *A. membranaceus* and *R. trichosanthis*. B16-F10 melanoma cell injection caused small black tumor spots (>2 mm in diameter) that appeared at the injection sites of cancer-injected mice after 2 days. Tumor weight in the herb extract-administered group decreased significantly, and a large area of tumor tissues disappeared. Herb extract caused the upregulation of E-cadherin and downregulation of N-cadherin, decreased LDH activity, decreased cell damage indicator malondialdehyde content, and decreased the activity of SOD, as well as reduced Akt protein expression in melanoma tissues, indicating the efficacy of the extracts in inhibiting the induced tumors [60].

In contrast, Burke et al. (2021) [54] found that TA-65 supplementation did not significantly alter the development or progression of UV-induced skin cancer in SKH1 hairless mice. No significant differences in tumor incidence, number, or overall burden were observed between TA-65-treated and control groups. Although a transient increase in larger tumors was observed at isolated time points, these differences were not statistically conclusive. Burke et al. suggested that TA-65 did not promote UV-induced skin cancer nor posed safety measures in this experimental model [54]. B16-OVA melanoma-bearing mice were used to evaluate the effects of *Astragalus* injection combined with the immunotherapy drug anti-PD-1. Astragalus injection antagonized the therapeutic efficacy of anti-PD-1. The combination, however, led to a significant reduction in tumor growth. Notably, there was a decrease in the infiltration of immune cells, including dendritic cells, CD4^+^ T cells, and CD8^+^ T cells. The treatment significantly downregulated MHC-II expression in both dendritic cells and B16-OVA cells. This downregulation was associated with decreased expression of class II trans-activator (CIITA), a key transcriptional activator of MHC-II genes, and reduced phosphorylation of STAT1, a critical component of the JAK/STAT signaling pathway (Table 3) [62].

Many authors mentioned several important limitations for TA-65 use. In an excellent metanalysis, Su et al. (2025) concluded that variability in telomere measurement methods and the short follow-up duration (median of 12 months) limited the ability to evaluate long-term cancer risks [28,63]. Additionally, none of the included studies accounted for changes in immune cell composition, such as shifts in naïve (immature white blood cells that developed in the thymus) versus memory T-cell ratios. Since leukocyte distribution can influence average telomere length in bulk samples, it was suggested that telomere elongation may reflect, in part, altered cell populations rather than genuine within-cell telomere extension [63]. Finally, the dose–response analysis in some studies was constrained by an uneven distribution of studies, with only one trial in the highest dose category. However, supplementary analyses (meta-regression and alternative grouping) consistently indicated no significant dose–response relationship [63]. Nevertheless, these authors recommended that clinicians could restrict the use of TA-65 to older patients (>60 years) with biomarker-confirmed immunosenescence along with strict stopping rules. They also recommend extending safety monitoring beyond 5 years. Overall, current clinical evidence remains limited, highlighting the need for larger, well-designed independent trials to confirm these findings.

## 6. Pharmacokinetics of *A. membranaceus*

### 6.1. Absorption, Metabolism, and Bioavailability

Many pilot studies indicated that about 50% of astragaloside IV may undergo metabolism, since only about 50% of parent astragaloside IV is recoverable in both the urine and feces [64,65]. Astragaloside IV extracted from *A. membranaceus* showed moderate to rapid elimination and linear pharmacokinetic behavior in the experimental animals across the tested dose ranges. Interestingly, AS-IV is little metabolized by liver metabolism trials, rapidly absorbed, and widely distributed in tissue. AS-IV concentration is highest in the liver and kidney, followed by the lung, heart, and spleen [64,66].

Zhang et al. (2007) [67] proved that AS-IV is bound extensively to plasma proteins and that most of it forms associative patterns in dog plasma. These authors recommended that the binding to plasma protein should be taken into account when AS-IV is considered for clinical use together with other high protein-binding drugs [67].

To evaluate the intestinal absorption of astragaloside IV under different conditions, Gu et al. (2004) [68] tested two different solutions, a pure aqueous solution of astragaloside IV and *Radix Astragali* oral solution, on two different groups of rats: bile duct-ligated rats (to assess the role of bile in absorption) and non-ligated rats (normal bile secretion). It was found that the absorption rate of astragaloside IV from the aqueous solution was significantly lower than from the Radix Astragali oral solution, and the absorption rate was lower in bile duct-ligated rats compared to non-ligated rats [68]. Another study indicated that AS-IV has low gastrointestinal tract absorption; the bioavailability of AS-IV in rats was in the range of 2.2–3% and in dogs it was 7.4% [66].

P-glycoprotein is an efflux transporter that pumps drugs back into the intestinal lumen. Gu et al. (2004) studied the effect of P-glycoprotein on astragaloside IV showing that the uptake was unaffected by P-glycoprotein inhibitors, and they found that most of the orally administered astragaloside IV was not absorbed or lost during first-pass metabolism [68].

In summary, astragaloside IV exhibits moderate pharmacokinetic properties characterized by rapid absorption, wide tissue distribution, and relatively fast elimination, with highest concentrations in the liver and kidneys. However, its oral bioavailability is low due to limited gastrointestinal absorption and partial metabolism. Additionally, its strong binding to plasma proteins and dependence on factors such as bile for absorption highlight important considerations for its clinical use and potential drug interactions.

### 6.2. Toxicokinetics

*A. membranaceus* and its main compound have been tested in both adult animals and pregnant animals. With the administration of AS-IV (0.5–1.0 mg/Kg) during pregnancy in Sprague Dawley rats and New Zealand rabbits, maternal toxicity and fetotoxicity were noticed, while teratogenicity was not observed. Further studies indicated that maternal exposure to AS-IV (1.0 mg/Kg, for 4 weeks) directly led to the delay of fur formation, eye opening, and cliff parry reflex (neurodevelopmental marker), while it had no effect on memory and learning of newborn rats [66]. Therefore, it was speculated that AS-IV could be administered with caution to pregnant women.

During a 7-day period of acute toxicity evaluation, BALB/c mice of both genders that were orally administered a water-soluble astragaloside IV derivative (astragalosidic acid) at a single dose of 5000 mg/Kg showed no overt signs of distress, and there were no observable symptoms of either toxicity or deaths [69]. In an earlier study, rats and beagles received APS and AS for 90 days without significant toxicity, even at doses 70- and 35-times higher than the human dose, respectively [70]. In animals, *A. membranaceus* and its main bioactive compounds are safe at standard doses in both short- and long-term toxicity tests, showing no significant side effects. However, high doses or prolonged use should be approached with caution as they may cause toxicity to other organs and biochemical parameters that have not been evaluated.

In humans, China’s health authorities officially considered *A. membranaceus* as generally recognized as safe (GRAS) status on 9 November 2023 and as an edible Chinese medicinal substance. However, preclinical and clinical studies reported some side effects of *A. membranaceus*. These are uncommon and mild, mainly including rash, itching, nasal symptoms, stomach upset, and diarrhea [70,71]. Also, very high doses may suppress the immune system. Hence, patients should avoid using Astragalus if they are taking immune-suppressing drugs. If a person has an immune system disease, such as multiple sclerosis, lupus, rheumatoid arthritis, or another condition known as an autoimmune disease, that person should not use Astragalus root. Because there are no long-term clinical studies, pregnant or nursing women should not use Astragalus root. As with any herbal supplement, it is always necessary to check with an appropriate healthcare provider before taking Astragalus root [31].

In summary, *A. membranaceus* and its main compounds, particularly astragaloside IV, are generally considered safe in animal studies at standard doses, showing minimal toxicity in both short- and long-term use. However, evidence of maternal and developmental effects at certain doses suggests caution during pregnancy. While officially recognized as safe for human use, mild side effects such as gastrointestinal discomfort and allergic reactions have been reported, and high doses may suppress immune function. Therefore, careful use is recommended, especially in pregnant women, those with autoimmune conditions, or patients taking immunosuppressive therapies.

## 7. Conclusions

*Astragalus membranaceus* is a Chinese medicinal herb characterized by its high phytochemical content such as cycloastragenol, astragaloside IV, and polysaccharides. It has multifaceted pharmacological effects that support the role of these compounds in anti-aging, regenerative, and anticancer interventions. The plant and its compounds have been claimed to cause telomerase activation, antioxidant pathways, and modulation of apoptotic signaling, thereby promoting cellular longevity, improving tissue function, and reducing senescence in multiple cell types, including dermal fibroblasts, neuronal cells, osteoblasts, and nucleus pulposus cells. Clinical and preclinical studies demonstrated that *A. membranaceus* can lengthen telomeres, improve skin health, mitigate age-related bone loss, and enhance regenerative capacity without increasing cancer risk.

In the context of oncology, saponins and polysaccharides have antiproliferative, apoptotic, and immunomodulatory effects against various types of cancer, including osteosarcoma and melanoma, through mechanisms involving caspase-dependent apoptosis, microRNA regulation, Akt signaling, and cytotoxic T-cell activation. However, some studies suggest potential interactions with immunotherapy, underscoring the need for careful evaluations in combination cancer therapies.

Overall, *A. membranaceus* represents a promising natural agent for promoting healthy aging, tissue regeneration, and cancer modulation, with a reasonably characterized safety profile. Nevertheless, many more clinical experiments are needed to identify dosages, clarify the long-term effects, and detect its role alongside traditional treatments.

## Figures and Tables

**Table 1:** Summary of the phytochemicals reported in *Astragalus membranaceus* (Fisch.).

Phytochemicals Class	Compounds Identified	Biological Relevance
Saponins (astragalosides)	astragaloside I, astragaloside II, astragaloside III, and astragaloside IV [1,15].	Telomerase activation, antioxidant, anti-inflammatory, IL-2-inducing activity, and anticancer activity [16].
Flavonoids/isoflavones	formononetin, calycosin, ononin, and calycosin-7-O-β-D-glucoside [1,15].	Estrogenic activity, antioxidant, anti-inflammatory: reduce iNOS, COX-2, IL-6, IL-1β, interferon-α, NO, NF-κB expression [17], free-radical scavenging [18], protect against superoxide anions [19], and improve antioxidant enzymes activity [20].
Amino acids	L-canavanine, asparagine, aspartic acid, glutamic acid, leucine, phenylalanine, alanine, proline, and arginine [1,15].	Nutritional and metabolic support.
Trace elements	scandium, chromium, cobalt, copper, selenium, molybdenum, cesium, iron, manganese, zinc, and rubidium [21].	Metabolic support and essential minerals.
Polysaccharides	water-soluble heteropolysaccharides, acid heteropolysaccharides, such as glucuronic acid, and galacturonic acid [1].	Immunomodulation, osteoprotective, and antihyperglycemic: reduce blood sugar, triglycerides, LDL, and insulin resistance [22].

**Table 2:** Summary of the effect of *A. membranaceus* root extract or its bioactive compounds on postmenopausal bone diseases and aged skin.

Compounds	Effect on Skin	Effect on Bone Diseases
Cycloastragenol	Removed senescent cells and improved skin rejuvenation potential by increasing apoptosis of senescent cells [32]. Reduced wrinkles and improved skin texture by stimulating extracellular matrix components like hyaluronic acid [44].	Prevented age-related bone loss, improved bone microarchitecture, and enhanced bone strength [39]. Likely supported anti-aging and bone-protective effects through modulation of the NRF2/ARE pathway, telomerase-related activity, and proteasome regulation [34].
Astragaloside	Protected against UV-induced collagen breakdown by downregulating MMP-1 expression and inhibiting collagen degradation [45].	Protected intervertebral disc cells, suggesting the prevention of disc degeneration and bone-related tissue aging by activating telomerase [14].
*A. membranaceus* root extract	Protected against UV-induced damage by enhancing telomerase activity [31]. Improved skin hydration, elasticity, and firmness; reduced wrinkles by stimulating collagen synthesis [46]. Protected dermal fibroblasts from photoaging by suppressing NF-κB activity [47].	Improved bone mineral density and enhanced bone metabolism [40,41]. Improved bone strength and density, and prevented osteoporosis by regulating the vitamin D/FGF23/Klotho pathway [41].
Astragalus polysaccharide	Promoted stem cell differentiation, which potentially regenerates skin and promotes wound healing by miR-760 inhibition and increasing ANKFY1 expression [37].	Reduced alveolar bone destruction, suppressed local osteoclastogenesis, and protected periodontal bone [38]. Supported bone regeneration potential by ANKFY1 upregulation via miR-760 inhibition [37].

**Table 3:** Anticancer effects of *Astragalus membranaceus* and its compounds on skin and bone.

Compound	Skin Cancer Model	Bone Cancer Model
Astragaloside IV	Decreased proliferation of vulvar squamous carcinoma cells by upregulating P53, P21, cyclin D1, Bax, and cC-3 [51].	Increased osteosarcoma cell apoptosis through the regulation of caspase-dependent Fas/FasL signaling [57].
Astragalus polysaccharides	Enhanced immune response in S180 tumor-bearing mice [56].	Increased apoptosis and decreased proliferation of osteosarcoma cells by the upregulation of miR-133a [58].
*A. membranaceus*	Reduced efficacy of anti-PD-1 in melanoma cells by decreasing the regulation of MHC-II expression [62].	Inhibited tumor growth of osteosarcoma by the activation of cytotoxic T lymphocytes [55].

## Data Availability

All data provided in this work were provided through a review of the available literature.
